# (*E*)-Methyl *N*′-[1-(2-hydroxy­phen­yl)ethyl­idene]hydrazinecarboxyl­ate

**DOI:** 10.1107/S1600536808024434

**Published:** 2008-08-13

**Authors:** Bin Zhu, Xiang-Wei Cheng

**Affiliations:** a Zhejiang Police College Experience Center, Zhejiang Police College, Hangzhou 310053, People’s Republic of China

## Abstract

The mol­ecule of the title compound, C_10_H_12_N_2_O_3_, adopts a *trans* configuration with respect to the C=N bond. The dihedral angle between the benzene ring and the hydrazinecarboxyl­ate plane is 8.98 (7)°. Intra­molecular O—H⋯N and C—H⋯N hydrogen bonds are observed. Mol­ecules are linked into chains along the *c* axis by N—H⋯O hydrogen bonds. In addition, C—H⋯π inter­actions are observed.

## Related literature

For general background, see: Parashar *et al.* (1988 ▶); Hadjoudis *et al.* (1987 ▶); Borg *et al.* (1999 ▶). For a related structure, see: Cheng (2008 ▶).
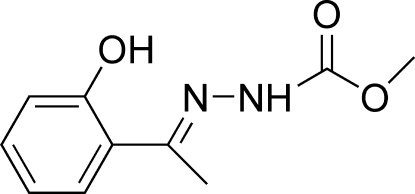


## Experimental

### 

#### Crystal data

C_10_H_12_N_2_O_3_
*M* *_r_* = 208.22
Monoclinic, 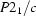

*a* = 8.6432 (8) Å
*b* = 12.6696 (11) Å
*c* = 9.9810 (9) Å
β = 109.837 (3)°
*V* = 1028.12 (16) Å^3^
*Z* = 4
Mo *K*α radiation
μ = 0.10 mm^−1^
*T* = 273 (2) K
0.28 × 0.24 × 0.23 mm

#### Data collection

Bruker SMART CCD area-detector diffractometer
Absorption correction: multi-scan (*SADABS*; Bruker, 2002 ▶) *T*_min_ = 0.973, *T*_max_ = 0.979
10601 measured reflections
1809 independent reflections
1587 reflections with *I* > 2σ(*I*)
*R*_int_ = 0.019

#### Refinement

*R*[*F* ^2^ > 2σ(*F* ^2^)] = 0.035
*wR*(*F* ^2^) = 0.107
*S* = 1.05
1809 reflections
140 parameters
H-atom parameters constrained
Δρ_max_ = 0.26 e Å^−3^
Δρ_min_ = −0.12 e Å^−3^

### 

Data collection: *SMART* (Bruker, 2002 ▶); cell refinement: *SAINT* (Bruker, 2002 ▶); data reduction: *SAINT*; program(s) used to solve structure: *SHELXS97* (Sheldrick, 2008 ▶); program(s) used to refine structure: *SHELXL97* (Sheldrick, 2008 ▶); molecular graphics: *SHELXTL* (Sheldrick, 2008 ▶); software used to prepare material for publication: *SHELXTL*.

## Supplementary Material

Crystal structure: contains datablocks I, global. DOI: 10.1107/S1600536808024434/ci2645sup1.cif

Structure factors: contains datablocks I. DOI: 10.1107/S1600536808024434/ci2645Isup2.hkl

Additional supplementary materials:  crystallographic information; 3D view; checkCIF report

## Figures and Tables

**Table 1:** Hydrogen-bond geometry (Å, °)

*D*—H⋯*A*	*D*—H	H⋯*A*	*D*⋯*A*	*D*—H⋯*A*
O1—H1⋯N1	0.82	1.85	2.5625 (14)	145
N2—H2*A*⋯O2^i^	0.86	2.25	3.0550 (14)	156
C8—H8*A*⋯N2	0.96	2.47	2.820 (2)	101
C8—H8*C*⋯*Cg*1^ii^	0.96	2.93	3.803 (2)	151

Symmetry codes: (i) 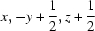; (ii) 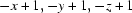. *Cg*1 is the centroid of the C1–C6 ring.

**Fig. 1.**
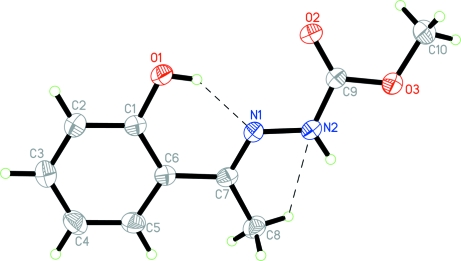
Molecular structure of the title compound, showing 30% probability displacement ellipsoids and the atomic numbering. Dashed lines indicate intramolecular hydrogen bonds.

**Fig. 2.**
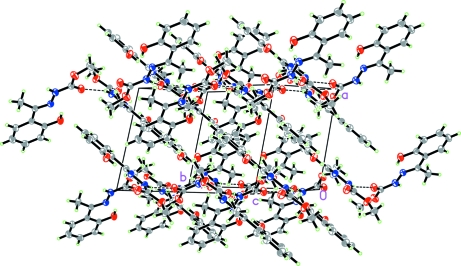
Crystal packing of the title compound. Dashed lines indicate intermolecular hydrogen bonds.

## supplementary crystallographic information

### Comment

Benzaldehydehydrazone compounds have received considerable attention for a long time due to their pharmacological activity (Parashar *et al.*, 1988) and their photochromic properties (Hadjoudis *et al.*, 1987). They are important intermediates of 1,3,4-oxadiazoles, which have been reported to be versatile compounds with many properties (Borg *et al.*, 1999). As a further investigation of this type of derivatives, the crystal structure of the title compound is reported here.

The title molecule (Fig.1) adopts a *trans* configuration with respect to the C═N bond. The C9/C10/N1/N2/O2/O3 plane of the hydrazine carboxylic acid methyl ester group is slightly twisted away from the attached ring. The dihedral angle between the C1–C6 ring and the C9/C10/N1/N2/O2/O3 plane is 8.98 (7)°. The bond lengths and angles agree with those observed for methyl *N*'-((*E*)-1-phenylethylidene)hydrazinecarboxylate (Cheng, 2008). Intramolecular O—H···N and C—H···N hydrogen bonds are observed.

The molecules are linked into chains along the *c* axis by N—H···O hydrogen bonds. In addition, C—H···π interactions are observed (Table 1, Fig.2).

### Experimental

2-Hydroxyacetophenone (1.36 g, 0.01 mol) and methyl hydrazinecarboxylate (0.9 g, 0.01 mol) were dissolved in stirred methanol (15 ml) and left for 2 h at room temperature. The resulting solid was filtered off and recrystallized from ethanol to give the title compound in 85% yield. Single crystals suitable for X-ray analysis were obtained by slow evaporation of an ethanol solution at room temperature (m.p. 465–467 K).

### Refinement

H atoms were positioned geometrically (O-H = 0.82 Å, N-H = 0.86 Å and C-H = 0.93 or 0.96 Å) and refined using a riding model, with *U*_iso_(H) = 1.2–1.5*U*_eq_(C). A rotating group model was used for the methyl groups.

### Crystal data

**Table:**

C_10_H_12_N_2_O_3_	*F*_000_ = 440
*M**_r_* = 208.22	*D*_x_ = 1.345 Mg m^−^^3^
Monoclinic, *P*2_1_/*c*	Mo *K*α radiation λ = 0.71073 Å
Hall symbol: -P 2ybc	Cell parameters from 1809 reflections
*a* = 8.6432 (8) Å	θ = 2.5–25.0º
*b* = 12.6696 (11) Å	µ = 0.10 mm^−^^1^
*c* = 9.9810 (9) Å	*T* = 273 (2) K
β = 109.837 (3)º	Block, colourless
*V* = 1028.12 (16) Å^3^	0.28 × 0.24 × 0.23 mm
*Z* = 4	

### Data collection

**Table:**

Bruker SMART CCD area-detector diffractometer	1809 independent reflections
Radiation source: fine-focus sealed tube	1587 reflections with *I* > 2σ(*I*)
Monochromator: graphite	*R*_int_ = 0.019
*T* = 273(2) K	θ_max_ = 25.1º
φ and ω scans	θ_min_ = 2.5º
Absorption correction: multi-scan(SADABS; Bruker, 2002)	*h* = −10→9
*T*_min_ = 0.973, *T*_max_ = 0.979	*k* = −14→13
10601 measured reflections	*l* = −11→11

### Refinement

**Table:**

Refinement on *F*^2^	Hydrogen site location: inferred from neighbouring sites
Least-squares matrix: full	H-atom parameters constrained
*R*[*F*^2^ > 2σ(*F*^2^)] = 0.035	*w* = 1/[σ^2^(*F*_o_^2^) + (0.0557*P*)^2^ + 0.2233*P*] where *P* = (*F*_o_^2^ + 2*F*_c_^2^)/3
*wR*(*F*^2^) = 0.107	(Δ/σ)_max_ = 0.001
*S* = 1.05	Δρ_max_ = 0.26 e Å^−^^3^
1809 reflections	Δρ_min_ = −0.12 e Å^−^^3^
140 parameters	Extinction correction: SHELXL97 (Sheldrick, 2008), Fc^*^=kFc[1+0.001xFc^2^λ^3^/sin(2θ)]^-1/4^
Primary atom site location: structure-invariant direct methods	Extinction coefficient: 0.017 (3)
Secondary atom site location: difference Fourier map	

### Special details

**Table:**

Geometry. All e.s.d.'s (except the e.s.d. in the dihedral angle between two l.s. planes) are estimated using the full covariance matrix. The cell e.s.d.'s are taken into account individually in the estimation of e.s.d.'s in distances, angles and torsion angles; correlations between e.s.d.'s in cell parameters are only used when they are defined by crystal symmetry. An approximate (isotropic) treatment of cell e.s.d.'s is used for estimating e.s.d.'s involving l.s. planes.
Refinement. Refinement of *F*^2^ against ALL reflections. The weighted *R*-factor *wR* and goodness of fit *S* are based on *F*^2^, conventional *R*-factors *R* are based on *F*, with *F* set to zero for negative *F*^2^. The threshold expression of *F*^2^ > σ(*F*^2^) is used only for calculating *R*-factors(gt) *etc*. and is not relevant to the choice of reflections for refinement. *R*-factors based on *F*^2^ are statistically about twice as large as those based on *F*, and *R*- factors based on ALL data will be even larger.

### Fractional atomic coordinates and isotropic or equivalent isotropic displacement parameters (Å^2^)

**Table:**

	*x*	*y*	*z*	*U*_iso_*/*U*_eq_	
C1	0.62350 (16)	−0.02927 (10)	0.76460 (14)	0.0409 (3)	
C6	0.67306 (16)	−0.04195 (10)	0.91401 (14)	0.0403 (3)	
C7	0.79166 (16)	0.03028 (10)	1.01395 (13)	0.0404 (3)	
C9	1.01051 (17)	0.25416 (11)	0.97214 (14)	0.0430 (3)	
C2	0.51172 (17)	−0.09885 (12)	0.67429 (16)	0.0497 (4)	
H2	0.4799	−0.0896	0.5762	0.060*	
C3	0.44767 (19)	−0.18132 (12)	0.72853 (18)	0.0577 (4)	
H3	0.3740	−0.2279	0.6671	0.069*	
C5	0.60373 (19)	−0.12639 (12)	0.96486 (17)	0.0538 (4)	
H5	0.6335	−0.1365	1.0627	0.065*	
C4	0.4927 (2)	−0.19499 (14)	0.87417 (19)	0.0623 (5)	
H4	0.4484	−0.2502	0.9109	0.075*	
C10	1.1944 (2)	0.39775 (14)	1.01329 (19)	0.0650 (5)	
H10A	1.2220	0.3668	0.9365	0.098*	
H10B	1.2933	0.4178	1.0883	0.098*	
H10C	1.1272	0.4591	0.9794	0.098*	
C8	0.8508 (2)	0.00907 (13)	1.17083 (15)	0.0595 (4)	
H8A	0.9553	0.0427	1.2149	0.089*	
H8B	0.8622	−0.0657	1.1871	0.089*	
H8C	0.7727	0.0365	1.2110	0.089*	
O1	0.68111 (13)	0.04936 (8)	0.70165 (10)	0.0535 (3)	
H1	0.7464	0.0857	0.7631	0.080*	
O2	0.98628 (13)	0.25492 (8)	0.84561 (10)	0.0539 (3)	
O3	1.10557 (14)	0.32236 (9)	1.06669 (11)	0.0603 (3)	
N1	0.83941 (13)	0.10946 (9)	0.95665 (11)	0.0416 (3)	
N2	0.94739 (14)	0.18236 (10)	1.04034 (11)	0.0470 (3)	
H2A	0.9736	0.1824	1.1315	0.056*	

### Atomic displacement parameters (Å^2^)

**Table:**

	*U*^11^	*U*^22^	*U*^33^	*U*^12^	*U*^13^	*U*^23^
C1	0.0454 (7)	0.0377 (7)	0.0412 (7)	0.0050 (5)	0.0167 (6)	0.0020 (5)
C6	0.0439 (7)	0.0363 (7)	0.0421 (7)	0.0051 (5)	0.0167 (6)	0.0023 (5)
C7	0.0460 (7)	0.0404 (7)	0.0361 (7)	0.0071 (6)	0.0156 (6)	0.0029 (5)
C9	0.0477 (8)	0.0444 (8)	0.0381 (7)	−0.0012 (6)	0.0163 (6)	−0.0035 (6)
C2	0.0521 (8)	0.0504 (9)	0.0447 (8)	0.0007 (6)	0.0138 (6)	−0.0056 (6)
C3	0.0532 (9)	0.0511 (9)	0.0655 (10)	−0.0078 (7)	0.0156 (8)	−0.0084 (7)
C5	0.0632 (9)	0.0506 (9)	0.0495 (8)	−0.0015 (7)	0.0216 (7)	0.0089 (7)
C4	0.0659 (10)	0.0522 (9)	0.0710 (11)	−0.0131 (7)	0.0260 (8)	0.0056 (8)
C10	0.0688 (10)	0.0671 (11)	0.0637 (10)	−0.0237 (9)	0.0284 (8)	−0.0088 (8)
C8	0.0833 (11)	0.0526 (9)	0.0384 (8)	−0.0026 (8)	0.0151 (7)	0.0045 (7)
O1	0.0721 (7)	0.0501 (6)	0.0362 (5)	−0.0107 (5)	0.0155 (5)	0.0007 (4)
O2	0.0721 (7)	0.0544 (7)	0.0368 (6)	−0.0094 (5)	0.0206 (5)	−0.0010 (4)
O3	0.0716 (7)	0.0673 (7)	0.0464 (6)	−0.0274 (6)	0.0258 (5)	−0.0137 (5)
N1	0.0460 (6)	0.0423 (6)	0.0358 (6)	−0.0029 (5)	0.0130 (5)	−0.0021 (5)
N2	0.0563 (7)	0.0524 (7)	0.0319 (6)	−0.0097 (5)	0.0145 (5)	−0.0034 (5)

### Geometric parameters (Å, °)

**Table:**

C1—O1	1.3592 (16)	C5—C4	1.381 (2)
C1—C2	1.390 (2)	C5—H5	0.93
C1—C6	1.4141 (19)	C4—H4	0.93
C6—C5	1.4027 (19)	C10—O3	1.4364 (19)
C6—C7	1.4796 (19)	C10—H10A	0.96
C7—N1	1.2899 (17)	C10—H10B	0.96
C7—C8	1.4974 (18)	C10—H10C	0.96
C9—O2	1.2081 (16)	C8—H8A	0.96
C9—O3	1.3364 (17)	C8—H8B	0.96
C9—N2	1.3567 (17)	C8—H8C	0.96
C2—C3	1.377 (2)	O1—H1	0.82
C2—H2	0.93	N1—N2	1.3757 (16)
C3—C4	1.382 (2)	N2—H2A	0.86
C3—H3	0.93		
			
O1—C1—C2	116.62 (12)	C5—C4—C3	119.71 (15)
O1—C1—C6	122.91 (12)	C5—C4—H4	120.1
C2—C1—C6	120.46 (13)	C3—C4—H4	120.1
C5—C6—C1	117.03 (13)	O3—C10—H10A	109.5
C5—C6—C7	120.73 (12)	O3—C10—H10B	109.5
C1—C6—C7	122.23 (12)	H10A—C10—H10B	109.5
N1—C7—C6	115.77 (11)	O3—C10—H10C	109.5
N1—C7—C8	123.80 (13)	H10A—C10—H10C	109.5
C6—C7—C8	120.43 (12)	H10B—C10—H10C	109.5
O2—C9—O3	125.45 (13)	C7—C8—H8A	109.5
O2—C9—N2	124.98 (13)	C7—C8—H8B	109.5
O3—C9—N2	109.56 (11)	H8A—C8—H8B	109.5
C3—C2—C1	120.68 (14)	C7—C8—H8C	109.5
C3—C2—H2	119.7	H8A—C8—H8C	109.5
C1—C2—H2	119.7	H8B—C8—H8C	109.5
C2—C3—C4	120.09 (14)	C1—O1—H1	109.5
C2—C3—H3	120.0	C9—O3—C10	116.48 (11)
C4—C3—H3	120.0	C7—N1—N2	120.41 (11)
C4—C5—C6	122.01 (14)	C9—N2—N1	116.81 (11)
C4—C5—H5	119.0	C9—N2—H2A	121.6
C6—C5—H5	119.0	N1—N2—H2A	121.6
			
O1—C1—C6—C5	179.88 (12)	C1—C6—C5—C4	0.4 (2)
C2—C1—C6—C5	−0.42 (19)	C7—C6—C5—C4	179.96 (14)
O1—C1—C6—C7	0.3 (2)	C6—C5—C4—C3	0.2 (3)
C2—C1—C6—C7	−179.99 (12)	C2—C3—C4—C5	−0.8 (3)
C5—C6—C7—N1	−175.42 (12)	O2—C9—O3—C10	−4.6 (2)
C1—C6—C7—N1	4.12 (19)	N2—C9—O3—C10	174.30 (13)
C5—C6—C7—C8	5.3 (2)	C6—C7—N1—N2	178.87 (11)
C1—C6—C7—C8	−175.19 (13)	C8—C7—N1—N2	−1.8 (2)
O1—C1—C2—C3	179.57 (13)	O2—C9—N2—N1	−4.4 (2)
C6—C1—C2—C3	−0.2 (2)	O3—C9—N2—N1	176.72 (11)
C1—C2—C3—C4	0.8 (2)	C7—N1—N2—C9	170.54 (12)

### Hydrogen-bond geometry (Å, °)

**Table:**

*D*—H···*A*	*D*—H	H···*A*	*D*···*A*	*D*—H···*A*
O1—H1···N1	0.82	1.85	2.5625 (14)	145
N2—H2A···O2^i^	0.86	2.25	3.0550 (14)	156
C8—H8A···N2	0.96	2.47	2.820 (2)	101
C8—H8C···Cg1^ii^	0.96	2.93	3.803 (2)	151

Symmetry codes: (i) *x*, −*y*+1/2, *z*+1/2; (ii) −*x*+1, −*y*+1, −*z*+1.
